# Identification and genotyping of feline infectious peritonitis-associated single nucleotide polymorphisms in the feline interferon-γ gene

**DOI:** 10.1186/1297-9716-45-57

**Published:** 2014-05-21

**Authors:** Li-En Hsieh, Ling-Ling Chueh

**Affiliations:** 1 Graduate Institute of Veterinary Medicine, School of Veterinary Medicine, National Taiwan University, Taipei 10617, Taiwan

## Abstract

Feline infectious peritonitis (FIP) is an immune-mediated, highly lethal disease caused by feline coronavirus (FCoV) infection. Currently, no protective vaccine or effective treatment for the disease is available. Studies have found that some cats survive the challenge of virulent FCoV isolates. Since cellular immunity is thought to be critical in preventing FIP and because diseased cats often show a significant decrease in interferon-γ (IFN-γ) production, we investigated whether single nucleotide polymorphisms (SNP) in the feline IFN-γ gene (*fIFNG*) are associated with the outcome of infection. A total of 82 asymptomatic and 63 FIP cats were analyzed, and 16 SNP were identified in intron 1 of *fIFNG*. Among these SNP, the *fFING* + *428 T* allele was shown to be a FIP-resistant allele (*p* = 0.03), and the heterozygous genotypes *01C/T* and *+408C/T* were found to be FIP-susceptible factors (*p* = 0.004). Furthermore, an *fIFNG* + *428* resistant allele also showed a clear correlation with the plasma level of IFN-γ in FIP cats. For the identification of these three FIP-related SNP, genotyping methods were established using amplification refractory mutation system PCR (ARMS-PCR) and restriction fragment length polymorphisms (RFLP), and the different genotypes could easily be identified without sequencing. The identification of additional FIP-related SNP will allow the selection of resistant cats and decrease the morbidity of the cat population to FIP.

## Introduction

Feline infectious peritonitis (FIP) is an immune-mediated disease caused by feline coronavirus (FCoV) infection. Despite the vast number of studies conducted since the recognition of FIP [1], neither effective vaccines nor therapeutic agents are available for the prevention or treatment of this often fatal disease. Therefore, it remains one of the most important feline infectious diseases.

Despite the ubiquitous existence of FCoV in cat populations around the world, the development of FIP was only observed in fewer than 5% of the FCoV-infected cats [2]. In addition, during the past few decades, several studies were conducted using various FCoV isolates, and regardless of the dose of virulent FCoV, some cats survived from the experimental infection [2-11]. Genetic polymorphisms in the host were suggested to be important in the resistance to FIP [2]; however, no specific gene with a clear correlation to resistance to this disease has ever been identified.

The protective immunity to FIP is thought to result mainly from cell-mediated immunity (CMI), and changes in the expression of several cytokines have been observed in cats with either experimentally induced or naturally occurring FIP [2]. The expression of one of the cytokines studied, interferon-γ (IFN-γ), was consistently decreased in diseased animals, and this gene is thought to play a protective role in the pathogenesis of FIP, since it is a key cytokine in CMI [6,8,12-14].

In recent decades, single nucleotide polymorphisms (SNP) in the IFN-γ gene (*IFNG*) have been found to be associated with various pathological conditions in humans [15] as well as in ruminants and fowl [16-23]. Nevertheless, this gene has not been investigated in cats. To identify a possible association between feline *IFNG* (*fIFNG*) SNP and the outcome of FCoV infection, some regions of the *fIFNG* gene were sequenced and analyzed. Three SNP with statistical relevance were found to be associated with the occurrence of FIP, and polymerase chain reaction (PCR) assays based on these differences were designed as a potential screening test for the selection of FIP-resistant populations.

## Materials and methods

### Specimens

Whole blood and buccal swabs were collected from 82 FCoV-infected asymptomatic cats and 64 FIP cats from 2005 to 2012 at the National Taiwan University Animal Hospital for the association analysis. All asymptomatic healthy cats were three years old or younger and had positive reverse transcription-nested PCR test results for FCoV infection [24] in any of the following samples upon first arriving at the hospital: whole blood or nasal, oral, conjunctival, or rectal swabs. In addition, these cats showed no FIP-related symptoms when recruited into the study, and except for 12 cats, they stayed healthy for at least two years. All the FIP cases enrolled in this study were cats showing typical clinical signs of FIP and further confirmed by necropsy, histopathological examinations and FCoV detection [24] in disease-associated tissues, i.e. body effusions, kidney, liver, spleen, mesenteric lymph node, lung, and/or brain.

To elucidate the role of host genetic background in the development of FIP, two viral pathogens, i.e. feline immunodeficiency virus (FIV) and feline leukemia virus (FeLV), that cause immunosuppression in cats were checked in FIP cats using nested PCR [25,26], and the positive cats were omitted from the association analysis.

### Identification of SNP in the partial fIFNG sequences

The genomic DNA from each cat was extracted using a genomic DNA mini kit (Geneaid Biotech, New Taipei City, Taiwan), and partial *fIFNG* sequences were amplified by PCR. Briefly, the genomic DNA was amplified with primers aligning to either the 5′ proximal regulatory region and intron 1 or the 5′ untranslated region and exon 2 of *fIFNG* (Table 1), and the PCR products were then sequenced from both ends using an auto sequencer ABI 3730XL (Applied Biosystems, San Mateo, USA). The sequences were aligned by Geneious 3.8.5 (Biomatters, Auckland, New Zealand), and the polymorphisms were identified.

**Table 1:** Primers used for the identification of SNP, ARMS-PCR and RFLP

**Primer**	**Orientation**^**a**^	**Position**^**b**^	**Sequence (5′ - 3′)**^**c**^	**T**_**A **_^**d**^	**Amplicon size**
**Identification of SNP**					
5′-PRR^e^ - intron 1	F	−677 ~ -654	CAGGGCAATGCAAAGCTGTGGTAG	65°C	1306 bp
	R	+629 ~ +607	GCGGCAGTAGAACTTTGAAACCA		
5′-UTR^f^ - exon 2	F	−44 ~ -25	CGGAGCTACTGATTTCAACT	63°C	1434 bp
	R	+1390 ~ +1371	GGAAAGAGGTAAGCTGGGTA		
**Genotyping**^**g**^					
+401	F (universal)	+320 ~ +341	GGGGCATTCATCAGTCTTCCAG	56°C	200 bp
	R (universal)	+519 ~ +500	AAGGTCAGGGTTAGCATGAA		
	F (*T* allele)	+382 ~ +402	TAATTTTGTGGTGAGAATC*T*A		138 bp
	R (*C* allele)	+418 ~ +400	CAACATCACAGTCTAAT*G*G		99 bp
+408	F	+320 ~ +341	GGGGCATTCATCAGTCTTCCAG	56°C	200 bp
	R	+519 ~ +500	AAGGTCAGGGTTAGCATGAA		
+428	F (universal)	+288 ~ +307	TACCCTCTGCTCAACTTGCT	67°C	232 bp
	R (universal)	+519 ~ +500	AAGGTCAGGGTTAGCATGAA		
	F (*T* allele)	+408 ~ +429	CTGTGATGTTGGGTAGTGTG*T*C		112 bp
	R (*C* allele)	+449 ~ +427	GGCTAGTCATTGTTTCAATAG*G*C		162 bp

^a^*F*: forward; *R*: reverse.

^b^The nucleotide positions start from the first translation start point (+1).

^c^The italicizing indicates the target SNP.

^d^Annealing temperature.

^e^5′-proximal regulatory region.

^f^5′-untranslated region.

^g^PCR was done with a heating and cooling rate of 3 and 2 °C, respectively.

### Linkage disequilibrium (LD) test and association analysis

An LD test and the creation of an LD plot were performed using LD_2_SNPing v 2.0 (Department of Electronics Engineering, National Kaohsiung University of Applied Science, Kaohsiung, Taiwan) [27]. The associations between SNP and the outcome of FCoV infection were analyzed. A Fisher’s exact test value of *P* < 0.05 was considered to represent a significant association.

### Quantification of IFN-γ levels in the plasma samples of FIP cats

The plasma samples of FIP cats collected at the day of presentation were stored at −20 °C before use. The concentration of IFN-γ in the plasma samples was determined using an antigen capture ELISA (R & D system, McKinley Place NE, USA) following the procedure advised by the manufacturer’s instructions.

### Genotyping of SNP by tetra-primer amplification refractory mutation system PCR (ARMS-PCR) and restriction fragment length polymorphisms (RFLP)

For the genotyping of SNP using ARMS-PCR, the PCR reactions contained 1 μL of template DNA, each primer at 500 nM, 200 μM dNTP, 1.5 mM MgCl_2_, and 0.4 U Phusion DNA polymerase (Thermo Scientific, Waltham, USA) in a total volume of 20 μL with 1× Phusion HF buffer. The primers and PCR conditions are listed in Table 1. The PCR products were resolved in 2% agarose gels and photographed using an imaging system. The PCR for the genotyping of SNP with RFLP was carried out with the same protocol, and the PCR products were digested with *HpyCH4*III (New England Biolabs, Ipswich, USA) following the manufacturer’s instructions. The digested DNA was resolved in 2% agarose gels and photographed using an imaging system.

## Results

### Polymorphisms in fIFNG

**Figure 1:**
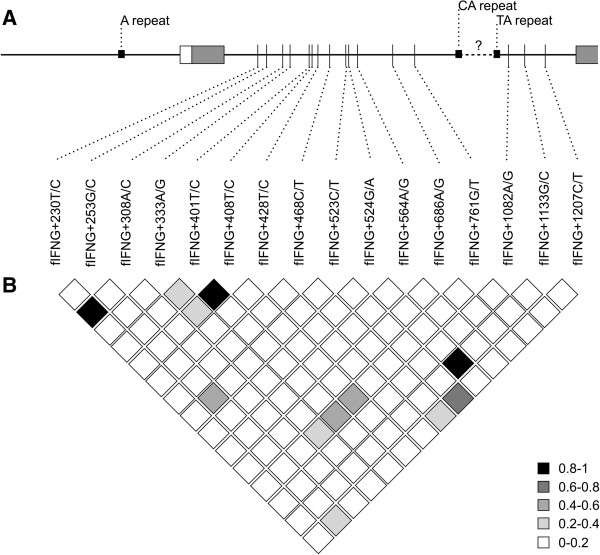
**A schematic of the partial *fIFNG *sequences analyzed in this study. (A)** A partial *fIFNG* sequence of 2067 bp was sequenced in this study, including the 5′ proximal regulatory region, 5′ UTR, exon 1, intron 1 and a partial exon 2 region. Black box: repeat region. White box: 5′ UTR. Gray boxes: exons 1 and 2. ?: a ~115 bp region between 2 repeat regions of unknown sequence. **(B)** LD plot of the 16 SNP tested in this study. The r^2^ values between different SNP are indicated by different colors.

Due to a lack of information on polymorphisms in *fIFNG*, DNA from 40 cats, including 20 asymptomatic and 20 FIP cats, was sequenced throughout the proximal regulatory region, the 5′-UTR, exon 1, intron 1 and a partial exon 2 region of *fIFNG*. In the 2067 bp analyzed, 3 repeat regions and 16 SNP were identified (Figure 1A). Among these 19 polymorphisms, only one was located in the proximal regulatory region, and the others were in intron 1. No polymorphisms were identified in the 5′-UTR, exon 1 or exon 2. Intron 1 was more polymorphic than the upstream region of *fIFNG*. The allele frequencies of all the SNP surveyed were identified and are listed in Figure 1. The mean allele frequencies of the minor alleles ranged from 4.8% to 47.6%.

The SNP were then subjected to LD analysis. An LD plot of all the SNP for the whole population, including asymptomatic and FIP cats, was generated (Figure 1B). Three pairs of SNP were found to be significantly associated (r^2^ > 0.8): *fIFNG + 230 T/C* and *fIFNG + 308A/C*, *fIFNG + 401 T/C* and *fIFNG + 408 T/C*, and *fIFNG + 524G/A* and *fIFNG + 1133G/C. fIFNG + 230* and *fIFNG + 308* and *fIFNG + 401* and *fIFNG + 408* had a high degree of LD and were inherited together 100% of the time.

### Association study

To elucidate the association between the identified SNP and the outcome of FCoV infection, the frequency of each genotype (Additional file 1) and allele (Additional file 2) was analyzed in 82 asymptomatic and 63 FIV and FeLV-free FIP cats. From all the SNP tested, only *fIFNG + 428C/T* was found to be significantly associated with the outcome of the infection. At position +428, there was a higher frequency of the *CT* genotype in asymptomatic control cats (19.5%) than in FIP cats (6.3%), and the data showed a significant correlation with disease resistance (*p* = 0.03) (Table 2). Similarly, the analysis of allele frequency and disease outcome also revealed that the *T* allele at position +428 was significantly associated with resistance to FIP (*p* = 0.03) (Table 2).

**Table 2:** **FIP-associated SNP in *fIFNG *identified in the whole cat population in this study**

**SNP**	**Control number (%)**	**FIP number (%)**	**OR (95% CI)**	***P***
***fIFNG + 428***				
*CC*	66 (80.5)	60 (93.8)	3.6 (1.2-11.5)	0.03
*CT*	16 (19.5)	4 (6.3)	Reference	
*TT*	0 (0.0)	0 (0.0)	…^a^	
Allele *C*	148 (90.2)	124 (96.9)	3.4 (1.1-10.3)	0.03
Allele *T*	16 (9.8)	4 (3.1)	Reference	

^a^no OR can be calculated.

Although both types I and II FCoV can cause FIP, type II FCoV has been found to be more related to acute infection [28] and can cause horizontal transmission [29] whereas infection with type I viruses often results in persistent infection [30], therefore the host genotype involved in type I FCoV infection likely influences the resistance to a greater extent. To gain a better insight into the effect of host genetic variation in *fIFNG* and FIP, 29 type I FCoV-infected FIP cats were selected for further analysis (Additional files 3 and 4). After analyzing the target population, despite no allele of any SNP showing an association with the infection outcome (Table 3), the heterozygous genotype (*CT* genotype) at positions +401 and +408 were found to be significantly associated with susceptibility to FIP (*p* = 0.004) (Table 3).

**Table 3:** **FIP-associated SNP in *fIFNG *identified in the type I FCoV-infected cats**

**SNP**	**Control number (%)**	**FIP number (%)**	**OR (95% CI)**	***P***
***fIFNG + 401***				
*TT*	43 (52.4)	11 (37.9)	0.4 (0.2-0.9)	0.004
*CT*	26 (31.7)	18 (62.1)	Reference	
*CC*	13 (15.9)	0 (0.0)	n/a^a^	
Allele *C*	112 (68.3)	40 (69.0)	…^b^	1.00
Allele *T*	52 (31.7)	18 (31.0)	…	
***fIFNG + 408***				
*TT*	43 (52.4)	11 (37.9)	0.4 (0.2-0.9)	0.004
*CT*	26 (31.7)	18 (62.1)	Reference	
*CC*	13 (15.9)	0 (0.0)	n/a^a^	
Allele *C*	112 (68.3)	40 (69.0)	…	1.00
Allele *T*	52 (31.7)	18 (31.0)	…	

^a^not available.

^b^no OR can be calculated.

### Production of IFN-γ in FIP cats carrying different genotypes

**Figure 2:**
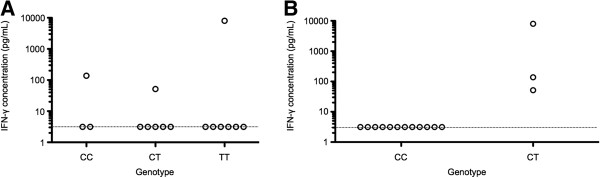
**Plasma concentration of IFN-γ in FIP cats carrying different genotypes at positions +401/+408 (A) and +428 (B).** The concentration of IFN-γ was measured using antigen capture ELISA. A dotted line indicates the detection limit of the assay.

To further validate the correlation between FIP-associated SNP and the production of IFN-γ, the concentration of IFN-γ in the plasma samples from 15 FIP cats was measured. For most of the FIP cats (12/15; 80%), the concentration was below the detection limit (<3.125 pg/mL) except for three cats. The concentrations of IFN-γ for cat 14, 17, and 69 were 52, 138 and > 8000 pg/mL, respectively (Figure 2, Additional file 5). These three animals were the only cats carrying the resistant allele (*T allele*) at position +428 whereas others were *fIFNG + 428CC* genotype (Figure 2B, Additional file 5). For the *fIFNG + 401/+408*, however, no clear correlation between the IFN-γ responses and genotypes was observed (Figure 2A, Additional file 5).

### Genotyping for disease-related SNP

**Figure 3:**
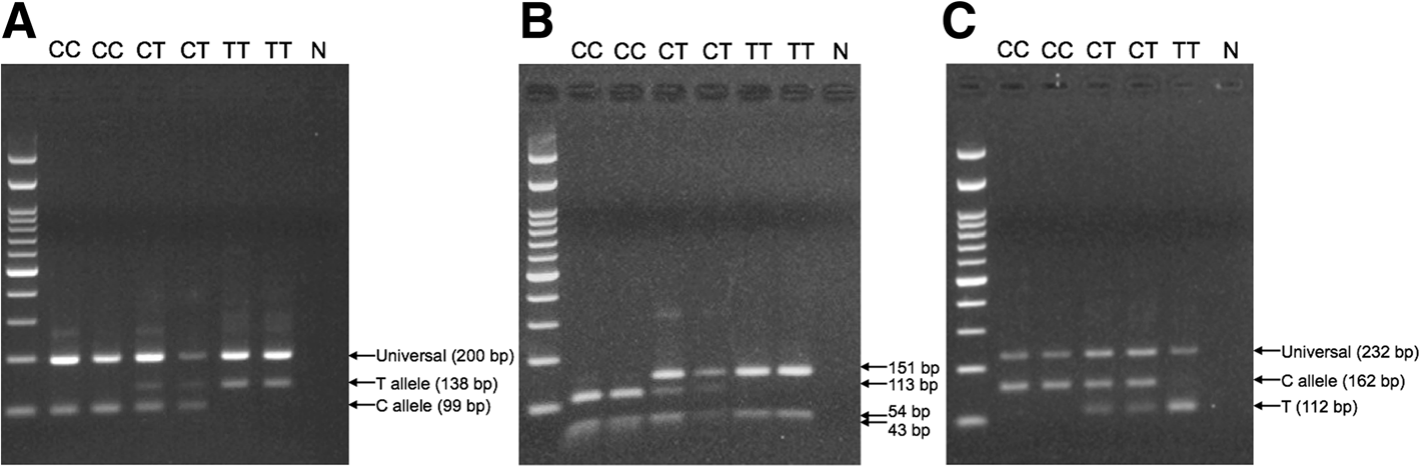
**The ARMS-PCR and RFLP established in this study for the genotyping at positions +401 (A), +408 (B) and +428 (C).** The primers and PCR conditions are listed in Table 1. The PCR products **(A and C)** and the RFLP products **(B)** were resolved in 2% agarose gel and photographed.

Through the association analysis and the quantification of plasma IFN-γ level, *fIFNG + 428C/T* was found to be associated with FIP and the plasma concentration of IFN-γ. In addition to this SNP, *fIFNG + 401C/T* and *fIFNG + 408C/T* were also shown to be associated with the type I FCoV-infected FIP population. To develop screening that can easily distinguish the resistant cats from the at-risk ones, ARMS-PCR specific for *fIFNG + 428C/T* and *fIFNG + 401C/T* was attempted. Using the tetra primer ARMS-PCR, the different alleles at position +401 could be successfully distinguished: universal control product: 200 bp; *T* allele: 138 bp; *C* allele: 99 bp (Figure 3A). Similarly, the *C* and *T* alleles could be determined at position +428: universal control product: 232 bp; *C* allele: 162 bp; *T* allele: 112 bp (Figure 3C). For *fIFNG + 408*, RFLP was used for genotyping, and the digested PCR products for the *T* allele could be found at 151 bp and 54 bp; the digested PCR products for the *C* allele could be found at 113 bp, 54 bp, and 43 bp (Figure 3B).

## Discussion

The occurrence of FIP is thought to be largely affected by viral virulence factors, and their roles in FIP have been intensely studied. Several viral genes, including *spike*[31-33], accessory gene *3c*[34-36] and 7b [37-39], and *membrane*[40], have been proposed to play important roles in the development of the disease in FCoV-infected cats. As a disease with immunopathogenesis entity, the knowledge of the host genetic factors that affect FIP is still very limited, and only two studies have reported such factors [41,42], in contrast to the considerable quantity of information on the virulence factors.

Addie et al. who conducted the first study, attempted to reveal the genetic background of the cats and their associations with the occurrence of FIP (Table 4) [42]. Considering its important role in the defense against viral infection, *feline leucocyte antigen (FLA)-DRB* polymorphisms were analyzed in four different domestic shorthaired (DSH) or other purebred populations: FIP, FCoV carrier, transient infection and resistant. However, no association was found between any of the *FLA-DRB* polymorphisms and the occurrence of FIP, which might be due to the small sample size (FIP: 8 cats; asymptomatic: 17 cats) used. To gain better insight, a larger number of cats in each population was used (FIP: 63 cats; asymptomatic: 82 cats), and the association was clearly observed in the present study (Table 4).

**Table 4:** Studies conducted on host genetic polymorphisms and their associations with FIP

**Target**	**Method**	**Breed**	**Grouping (number of cats)**	**Polymorphism associated with FIP**	**Reference**
*FLA-DRB*	1. Clonal sequence analysis	DSH^b^ and Pure breed	1. FIP (8)	Non	[42]
	2. RSCA^a^		2. Carrier (4)		
			3. Transient (10)		
			4. Resistant (3)		
Identified SNP	SNP gene chip	Birman cats	1. FIP (34)	1. A2.191286425 - *ELMO1*	[41]
			2. Healthy (151)	2. A1.196617776 - *ERAP1, 2*	
				3. A1.206840008 - *ERAP1, 2*	
				4. Un.59861682 - *ERAP1, 2*	
				5. E2.65509996 - UI ^c^	
*fIFNG*	PCR and sequencing	DSH and Pure breed	1. FIP (63)	1. *fIFNG + 401*	This study
			2. Healthy (82)	2. *fIFNG + 408*	
				3. *fIFNG + 428*	

^a^Reference strand mediated conformational analysis.

^b^Domestic shorthaired.

^c^Unidentified.

Recently, several FIP-associated SNP were identified by a massive screen of Birman cats using a commercialized SNP gene chip, and the candidate genes were located from 69 kb to more than 1 Mb away from the identified SNP (Table 4) [41]. In contrast to that report, the SNP were located within the candidate gene, *fIFNG*, in our study, which demonstrates a stronger linkage of the analyzed SNP to the candidate gene. In addition, the cat populations enrolled in this study, including approximately 50% DSH and another 50% pure breed or mixed breed cats, were more variable, and the SNP associated with the disease may have been better represented on the entire cat population.

IFN-γ is a crucial regulatory cytokine in CMI and is important for the control of intracellular pathogens. In FCoV infection, decreased IFN-γ production in FIP cats was consistently observed in multiple studies [6,8,12-14]. In addition, in FCoV-infected non-FIP cats, the peripheral blood mononuclear cells showed a significant increase in the IFN-γ response upon stimulation with the FCoV antigen when compared to the FIP cats [3]. The expression of IFN-γ was thought to protect against FCoV infection. In several studies on human viral infections, i.e., human immunodeficiency virus [43], hepatitis B and hepatitis C [44,45], a similar correlation between the magnitude of the IFN-γ response and disease manifestation was identified. The polymorphisms in *IFNG* were associated with the diseases caused by these viruses [46-48].

The SNP associated with FIP are located in the intron 1 region. Therefore, it is unlikely that they mediate the outcome of the infection directly by altering the function of IFN-γ. In humans, *IFNG* polymorphisms have been found that correlate with the expression of IFN-γ. In the promoter region, SNP at positions −183 and −155 were found to influence the binding of activating transcription factor-1 and the nuclear factor-associated T-cell site [49,50]. In addition, the number of *CA* microsatellite repeats was found to be tightly associated with the SNP at position +874 and to influence the production of IFN-γ by altering the binding activity of nuclear factor kappa-light-chain-enhancer of activated B cells [15]. In ewes, despite the polymorphisms in *INFG* at position -641, the microsatellite showed a significant effect on *IFNG* expression in the spleen, although the mechanism that mediates this effect remains unknown. In the present study, a clear correlation between the IFN-γ responses and the genotype at position +428 was observed (Figure 2B). The resistant *T* allele at position +428 may serve as a major factor to enhance the IFN-γ production upon infections of intracellular pathogens. Despite lacking correlation with the IFN-γ level, the SNP at positions +401 and +408 might still play a minor role in the alteration of the production of IFN-γ. Since the three FIP-related SNP identified in this study are located in a small cluster, it is possible that these polymorphisms alter the binding of one or more transcription factors and work together to influence the immune response to FCoV infection. However, the actual mechanism for the altered expression of IFN-γ remains to be investigated.

In this study, three SNP in *fIFNG* were found to be associated with the outcome of FCoV infection. Using the ARMS-PCR and RFLP tests established, the three SNP could be distinguished in the ordinary diagnostic laboratory without sequencing, and cats bearing either the susceptible or resistant genotypes could be identified. Since the disease outcome is usually influenced by multiple host genes, other candidate genes, i.e., tumor necrosis factor-α [51,52], interleukin-12 [6] and CD209 [53], are currently being surveyed. A combination of all the FIP-related SNP into a single genotyping microarray will allow the selection of resistant cats before breeding and eventually decrease the morbidity of the cat population to FIP.

## Competing interests

The authors declare that they have no competing interests.

## Authors’ contributions

LEH performed the sample preparation, *fIFNG* amplification and sequencing, measurement of the plasma concentration of IFN-γ, established genotyping methods and prepared the manuscript. LLC conceived the study, participated in the study design and coordination and contributed to the preparation of the manuscript. Both authors read and approved the final manuscript.

## Supplementary Material

Additional file 1 **Frequencies of the various genotypes and their associations with the outcome of FCoV infection.** The CT genotype at position +428 was significantly associated with the resistance of FIP. Click here for file

Additional file 2 **Frequencies of various alleles and their associations with the outcome of FCoV infection.** The T allele at position +428 was significantly associated with the resistance of FIP. Click here for file

Additional file 3 **Frequencies of various genotypes and their associations with the outcomes of type I FCoV infection.** The heterologous genotypes of *fIFNG* + *401* and *fIFNG* + *408* were associated with the susceptibility to FIP in type I FCoV-infected cats. Click here for file

Additional file 4 **Frequencies of various alleles and their associations with the outcomes of type I FCoV infection.** No allele of the analyzed SNP was associated with the occurrence of FIP in type I FCoV-infected cats. Click here for file

Additional file 5 **Concentration of IFN-γ in the plasma samples of FIP cats carrying different genotypes at positions + 401, +408, and +428 on *fIFNG*.** All the FIP cats carrying the CT genotype at position +428 were positive for the detection of plasma IFN-γ. Click here for file

## References

[B1] Holzworth J Some important disorders of cats Cornell Vet 1963 53 157 160 13961523

[B2] Pedersen NC A review of feline infectious peritonitis virus infection: 1963-2008 J Feline Med Surg 2009 11 225 258 10.1016/j.jfms.2008.09.008 19254859 PMC7129802

[B3] Satoh R Furukawa T Kotake M Takano T Motokawa K Gemma T Watanabe R Arai S Hohdatsu T Screening and identification of T helper 1 and linear immunodominant antibody-binding epitopes in the spike 2 domain and the nucleocapsid protein of feline infectious peritonitis virus Vaccine 2011 29 1791 1800 10.1016/j.vaccine.2010.12.106 21216312 PMC7115570

[B4] de Groot-Mijnes JD van Dun JM van der Most RG de Groot RJ Natural history of a recurrent feline coronavirus infection and the role of cellular immunity in survival and disease J Virol 2005 79 1036 1044 10.1128/JVI.79.2.1036-1044.2005 15613332 PMC538555

[B5] Haijema BJ Volders H Rottier PJ Live, attenuated coronavirus vaccines through the directed deletion of group-specific genes provide protection against feline infectious peritonitis J Virol 2004 78 3863 3871 10.1128/JVI.78.8.3863-3871.2004 15047802 PMC374255

[B6] Kiss I Poland AM Pedersen NC Disease outcome and cytokine responses in cats immunized with an avirulent feline infectious peritonitis virus (FIPV)-UCD1 and challenge-exposed with virulent FIPV-UCD8 J Feline Med Surg 2004 6 89 97 10.1016/j.jfms.2003.08.009 15123153 PMC7128844

[B7] Hohdatsu T Yamato H Ohkawa T Kaneko M Motokawa K Kusuhara H Kaneshima T Arai S Koyama H Vaccine efficacy of a cell lysate with recombinant baculovirus-expressed feline infectious peritonitis (FIP) virus nucleocapsid protein against progression of FIP Vet Microbiol 2003 97 31 44 10.1016/j.vetmic.2003.09.016 14637036 PMC7117512

[B8] Dean GA Olivry T Stanton C Pedersen NC In vivo cytokine response to experimental feline infectious peritonitis virus infection Vet Microbiol 2003 97 1 12 10.1016/j.vetmic.2003.08.010 14637034 PMC7117329

[B9] Wasmoen TL Kadakia NP Unfer RC Fickbohm BL Cook CP Chu HJ Acree WM Protection of cats from infectious peritonitis by vaccination with a recombinant raccoon poxvirus expressing the nucleocapsid gene of feline infectious peritonitis virus Adv Exp Med Biol 1995 380 221 228 10.1007/978-1-4615-1899-0_36 8830483

[B10] Vennema H de Groot RJ Harbour DA Horzinek MC Spaan WJ Primary structure of the membrane and nucleocapsid protein genes of feline infectious peritonitis virus and immunogenicity of recombinant vaccinia viruses in kittens Virology 1991 181 327 335 10.1016/0042-6822(91)90499-2 1847259 PMC7130817

[B11] Pedersen NC Boyle JF Floyd K Infection studies in kittens, using feline infectious peritonitis virus propagated in cell culture Am J Vet Res 1981 42 363 367 6267959

[B12] Gunn-Moore DA Caney SM Gruffydd-Jones TJ Helps CR Harbour DA Antibody and cytokine responses in kittens during the development of feline infectious peritonitis (FIP) Vet Immunol Immunopathol 1998 65 221 242 10.1016/S0165-2427(98)00156-1 9839876 PMC7120021

[B13] Giordano A Paltrinieri S Interferon-gamma in the serum and effusions of cats with feline coronavirus infection Vet J 2009 180 396 398 10.1016/j.tvjl.2008.02.028 18406642 PMC7110850

[B14] Gelain ME Meli M Paltrinieri S Whole blood cytokine profiles in cats infected by feline coronavirus and healthy non-FCoV infected specific pathogen-free cats J Feline Med Surg 2006 8 389 399 10.1016/j.jfms.2006.05.002 16777454 PMC7130096

[B15] Pravica V Perrey C Stevens A Lee JH Hutchinson IV A single nucleotide polymorphism in the first intron of the human IFN-gamma gene: absolute correlation with a polymorphic CA microsatellite marker of high IFN-gamma production Hum Immunol 2000 61 863 866 10.1016/S0198-8859(00)00167-1 11053629

[B16] Maryam J Babar ME Nadeem A Hussain T Genetic variants in interferon gamma (IFN-gamma) gene are associated with resistance against ticks in Bos taurus and Bos indicus Mol Biol Rep 2012 39 4565 4570 10.1007/s11033-011-1246-8 21960011

[B17] Pant SD Verschoor CP Skelding AM Schenkel FS You Q Biggar GA Kelton DF Karrow NA Bovine IFNGR2, IL12RB1, IL12RB2, and IL23R polymorphisms and MAP infection status Mamm Genome 2011 22 583 588 10.1007/s00335-011-9332-8 21597988

[B18] Verschoor CP Pant SD Biggar GA Schenkel FS Sharma BS Karrow NA Identification of SNPs in interferon gamma, interleukin-22, and their receptors and associations with health and production-related traits in Canadian Holstein bulls Anim Biotechnol 2011 22 7 15 10.1080/10495398.2011.536078 21328101

[B19] Dervishi E Uriarte J Valderrabano J Calvo JH Structural and functional characterisation of the ovine interferon gamma (IFNG) gene: its role in nematode resistance in Rasa Aragonesa ewes Vet Immunol Immunopathol 2011 141 100 108 10.1016/j.vetimm.2011.02.013 21419496

[B20] Darlay RJ McCarthy AJ Illot NE Smith JE Shaw MA Novel polymorphisms in ovine immune response genes and their association with abortion Anim Genet 2011 42 535 543 10.1111/j.1365-2052.2011.02180.x 21906104

[B21] Dukkipati VS Blair HT Garrick DJ Lopez-Villalobos N Whittington RJ Reddacliff LA Eppleston J Windsor P Murray A Association of microsatellite polymorphisms with immune responses to a killed Mycobacterium avium subsp. paratuberculosis vaccine in Merino sheep N Z Vet J 2010 58 237 245 10.1080/00480169.2010.69154 20927174

[B22] Downing T Lynn DJ Connell S Lloyd AT Bhuiyan AK Silva P Naqvi AN Sanfo R Sow RS Podisi B O’Farrelly C Hanotte O Bradley DG Contrasting evolution of diversity at two disease-associated chicken genes Immunogenetics 2009 61 303 314 10.1007/s00251-009-0359-x 19247647

[B23] Kramer J Malek M Lamont SJ Association of twelve candidate gene polymorphisms and response to challenge with Salmonella enteritidis in poultry Anim Genet 2003 34 339 348 10.1046/j.1365-2052.2003.01027.x 14510669

[B24] Herrewegh AA de Groot RJ Cepica A Egberink HF Horzinek MC Rottier PJ Detection of feline coronavirus RNA in feces, tissues, and body fluids of naturally infected cats by reverse transcriptase PCR J Clin Microbiol 1995 33 684 689 7751377 10.1128/jcm.33.3.684-689.1995 PMC228014

[B25] Nishimura Y Goto Y Pang H Endo Y Mizuno T Momoi Y Watari T Tsujimoto H Hasegawa A Genetic heterogeneity of env gene of feline immunodeficiency virus obtained from multiple districts in Japan Virus Res 1998 57 101 112 10.1016/S0168-1702(98)00085-9 9833889

[B26] Stiles J Bienzle D Render JA Buyukmihci NC Johnson EC Use of nested polymerase chain reaction (PCR) for detection of retroviruses from formalin-fixed, paraffin-embedded uveal melanomas in cats Vet Ophthalmol 1999 2 113 116 10.1046/j.1463-5224.1999.00066.x 11397251

[B27] Chang HW Chuang LY Chang YJ Cheng YH Hung YC Chen HC Yang CH LD2SNPing: linkage disequilibrium plotter and RFLP enzyme mining for tag SNPs BMC Genet 2009 10 26 19500380 10.1186/1471-2156-10-26 PMC2709117

[B28] Lin CN Su BL Wang CH Hsieh MW Chueh TJ Chueh LL Genetic diversity and correlation with feline infectious peritonitis of feline coronavirus type I and II: A 5-year study in Taiwan Vet Microbiol 2009 136 233 239 10.1016/j.vetmic.2008.11.010 19117699 PMC7117496

[B29] Wang YT Su BL Hsieh LE Chueh LL An outbreak of feline infectious peritonitis in a Taiwanese shelter: epidemiologic and molecular evidence for horizontal transmission of a novel type II feline coronavirus Vet Res 2013 44 57 10.1186/1297-9716-44-57 23865689 PMC3720556

[B30] Addie DD Schaap IA Nicolson L Jarrett O Persistence and transmission of natural type I feline coronavirus infection J Gen Virol 2003 84 2735 2744 10.1099/vir.0.19129-0 13679608

[B31] Rottier PJ Nakamura K Schellen P Volders H Haijema BJ Acquisition of macrophage tropism during the pathogenesis of feline infectious peritonitis is determined by mutations in the feline coronavirus spike protein J Virol 2005 79 14122 14130 10.1128/JVI.79.22.14122-14130.2005 16254347 PMC1280227

[B32] Chang HW Egberink HF Halpin R Spiro DJ Rottier PJ Spike protein fusion Peptide and feline coronavirus virulence Emerg Infect Dis 2012 18 1089 1095 10.3201/eid1807.120143 22709821 PMC3376813

[B33] Licitra BN Millet JK Regan AD Hamilton BS Rinaldi VD Duhamel GE Whittaker GR Mutation in spike protein cleavage site and pathogenesis of feline coronavirus Emerg Infect Dis 2013 19 1066 1073 10.3201/eid1907.121094 23763835 PMC3713968

[B34] Vennema H Poland A Foley J Pedersen NC Feline infectious peritonitis viruses arise by mutation from endemic feline enteric coronaviruses Virology 1998 243 150 157 10.1006/viro.1998.9045 9527924 PMC7131759

[B35] Chang HW de Groot RJ Egberink HF Rottier PJ Feline infectious peritonitis: insights into feline coronavirus pathobiogenesis and epidemiology based on genetic analysis of the viral 3c gene J Gen Virol 2010 91 415 420 10.1099/vir.0.016485-0 19889934

[B36] Hsieh LE Huang WP Tang DJ Wang YT Chen CT Chueh LL 3C protein of feline coronavirus inhibits viral replication independently of the autophagy pathway Res Vet Sci 2013 95 1241 1247 10.1016/j.rvsc.2013.08.011 24050534 PMC7111855

[B37] Herrewegh AA Vennema H Horzinek MC Rottier PJ de Groot RJ The molecular genetics of feline coronaviruses: comparative sequence analysis of the ORF7a/7b transcription unit of different biotypes Virology 1995 212 622 631 10.1006/viro.1995.1520 7571432 PMC7131361

[B38] Vennema H Rossen JW Wesseling J Horzinek MC Rottier PJ Genomic organization and expression of the 3′ end of the canine and feline enteric coronaviruses Virology 1992 191 134 140 10.1016/0042-6822(92)90174-N 1329312 PMC7131216

[B39] Takano T Tomiyama Y Katoh Y Nakamura M Satoh R Hohdatsu T Mutation of neutralizing/antibody-dependent enhancing epitope on spike protein and 7b gene of feline infectious peritonitis virus: influences of viral replication in monocytes/macrophages and virulence in cats Virus Res 2011 156 72 80 10.1016/j.virusres.2010.12.020 21211540 PMC7114493

[B40] Brown MA Troyer JL Pecon-Slattery J Roelke ME O’Brien SJ Genetics and pathogenesis of feline infectious peritonitis virus Emerg Infect Dis 2009 15 1445 1452 10.3201/eid1509.081573 19788813 PMC2819880

[B41] Golovko L Lyons LA Liu H Sorensen A Wehnert S Pedersen NC Genetic susceptibility to feline infectious peritonitis in Birman cats Virus Res 2013 175 58 63 10.1016/j.virusres.2013.04.006 23619280 PMC4342855

[B42] Addie DD Kennedy LJ Ryvar R Willoughby K Gaskell RM Ollier WE Nart P Radford AD Feline leucocyte antigen class II polymorphism and susceptibility to feline infectious peritonitis J Feline Med Surg 2004 6 59 62 10.1016/j.jfms.2003.12.010 15123149 PMC7129417

[B43] Bailer RT Holloway A Sun J Margolick JB Martin M Kostman J Montaner LJ IL-13 and IFN-gamma secretion by activated T cells in HIV-1 infection associated with viral suppression and a lack of disease progression J Immunol 1999 162 7534 7542 10358209

[B44] Guidotti LG Ando K Hobbs MV Ishikawa T Runkel L Schreiber RD Chisari FV Cytotoxic T lymphocytes inhibit hepatitis B virus gene expression by a noncytolytic mechanism in transgenic mice Proc Natl Acad Sci U S A 1994 91 3764 3768 10.1073/pnas.91.9.3764 8170985 PMC43662

[B45] Heim MH Innate immunity and HCV J Hepatol 2013 58 564 574 23063572 10.1016/j.jhep.2012.10.005

[B46] Qi S Cao B Jiang M Xu C Dai Y Li K Wang K Ke Y Ning T Association of the -183 polymorphism in the IFN-gamma gene promoter with hepatitis B virus infection in the Chinese population J Clin Lab Anal 2005 19 276 281 10.1002/jcla.20090 16302211 PMC6807984

[B47] Kang MW Pyo CW Wie SH Choi HB Kim SY Kim YK Lee JS Kim TG Associations of IFN-gamma polymorphism with HIV-1 infection in the Korean population AIDS Res Hum Retroviruses 2006 22 297 299 10.1089/aid.2006.22.297 16545018

[B48] Dai CY Chuang WL Hsieh MY Lee LP Hou NJ Chen SC Lin ZY Hsieh MY Wang LY Tsai JF Chang WY Yu ML Polymorphism of interferon-gamma gene at position +874 and clinical characteristics of chronic hepatitis C Transl Res 2006 148 128 133 10.1016/j.trsl.2006.04.005 16938650

[B49] Chevillard C Henri S Stefani F Parzy D Dessein A Two new polymorphisms in the human interferon gamma (IFN-gamma) promoter Eur J Immunogenet 2002 29 53 56 10.1046/j.0960-7420.2001.00281.x 11841489

[B50] Barbulescu K Meyer zum Buschenfelde KH Neurath MF Constitutive and inducible protein/DNA interactions of the interferon-gamma promoter in vivo in [corrected] CD45RA and CD45R0 T helper subsets Eur J Immunol 1997 27 1098 1107 10.1002/eji.1830270509 9174598

[B51] Takano T Hohdatsu T Toda A Tanabe M Koyama H TNF-alpha, produced by feline infectious peritonitis virus (FIPV)-infected macrophages, upregulates expression of type II FIPV receptor feline aminopeptidase N in feline macrophages Virology 2007 364 64 72 10.1016/j.virol.2007.02.006 17382365 PMC7103289

[B52] Takano T Hohdatsu T Hashida Y Kaneko Y Tanabe M Koyama H A “possible” involvement of TNF-alpha in apoptosis induction in peripheral blood lymphocytes of cats with feline infectious peritonitis Vet Microbiol 2007 119 121 131 10.1016/j.vetmic.2006.08.033 17046178 PMC7117258

[B53] Regan AD Ousterout DG Whittaker GR Feline lectin activity is critical for the cellular entry of feline infectious peritonitis virus J Virol 2010 84 7917 7921 10.1128/JVI.00964-10 20484511 PMC2897608

