# Supplementary material for: Identification and genotyping of feline infectious peritonitis-associated single nucleotide polymorphisms in the feline interferon-γ gene
Source: Vet Res. 2014 May 21;45(1):57. doi: 10.1186/1297-9716-45-57 (PMC4041894; doi:10.1186/1297-9716-45-57)
Supplement: Additional file 1 — Frequencies of the various genotypes and their associations with the outcome of FCoV infection. The CT genotype at position +428 was significantly associated with the resistance of FIP. [file 1297-9716-45-57-S1.docx]

**Additional file 1 Frequencies of the various genotypes and their associations with the outcome of FCoV infection.**

| SNP | Control  Number (%) | FIP  Number (%) | OR (95% CI) | *P* |
| --- | --- | --- | --- | --- |
| ***fIFNG+230*** |  |  |  |  |
| *TT* | 73 (89.0) | 54 (85.7) | ... | 0.69 |
| *CT* | 7 (8.5) | 8 (12.7) | ... |  |
| *CC* | 2 (2.4) | 1 (1.6) | ... |  |
| ***fIFNG+253*** |  |  |  |  |
| *GG* | 43 (52.4) | 27 (42.9) | ... | 0.47 |
| *GC* | 29 (35.4) | 25 (39.7) | ... |  |
| *CC* | 10 (12.2) | 11 (17.5) | ... |  |
| ***fIFNG+308*** |  |  |  |  |
| *AA* | 73 (89.0) | 54 (85.7) | ... | 0.69 |
| *AC* | 7 (8.5) | 8 (12.7) | ... |  |
| *CC* | 2 (2.4) | 1 (1.6) | ... |  |
| ***fIFNG+333*** |  |  |  |  |
| *AA* | 50 (61.0) | 39 (61.9) | ... | 1.0 |
| *AG* | 26 (31.7) | 19 (30.2) | ... |  |
| *GG* | 6 (7.3) | 5 (7.9) | ... |  |
| ***fIFNG+401*** |  |  |  |  |
| *TT* | 43 (52.4) | 24 (38.1) | ... | 0.07 |
| *CT* | 26 (31.7) | 32 (50.8) | ... |  |
| *CC* | 13 (15.9) | 7 (11.1) | ... |  |
| ***fIFNG+408*** |  |  |  |  |
| *TT* | 43 (52.4) | 24 (38.1) | ... | 0.07 |
| *CT* | 26 (31.7) | 32 (50.8) | ... |  |
| *CC* | 13 (15.9) | 7 (11.1) | ... |  |
| ***fIFNG+428*** |  |  |  |  |
| *CC* | 66 (80.5) | 59 (93.7) | 3.6 (1.1-11.3) | 0.03 |
| *CT* | 16 (19.5) | 4 (6.3) | Reference |  |
| *TT* | 0 (0.0) | 0 (0.0) | ... |  |
| ***fIFNG+468*** |  |  |  |  |
| *CC* | 30 (36.6) | 23 (36.5) | ... | 0.45 |
| *CT* | 40 (48.8) | 26 (41.3) | ... |  |
| *TT* | 12 (14.6) | 14 (22.2) | ... |  |
| ***fIFNG+523*** |  |  |  |  |
| *CC* | 34 (41.5) | 22 (34.9) | ... | 0.10 |
| *CT* | 29 (35.4) | 33 (52.4) | ... |  |
| *TT* | 19 (23.2) | 8 (12.7) | ... |  |
| ***fIFNG+524*** |  |  |  |  |
| *GG* | 66 (80.5) | 51 (81.0) | ... | 1.0 |
| *GA* | 14 (17.1) | 10 (15.9) | ... |  |
| *AA* | 2 (2.4) | 2 (3.2) | ... |  |
| ***fIFNG+564*** |  |  |  |  |
| *AA* | 71 (86.6) | 51 (81.0) | ... | 0.34 |
| *AG* | 8 (9.8) | 11 (17.5) | ... |  |
| *GG* | 3 (3.7) | 1 (1.6) | ... |  |
| ***fIFNG+686*** |  |  |  |  |
| *AA* | 75 (91.5) | 57 (90.5) | ... | 0.87 |
| *AG* | 6 (7.3) | 6 (9.5) | ... |  |
| *GG* | 1 (1.2) | 0 (0.0) | ... |  |
| ***fIFNG+761*** |  |  |  |  |
| *GG* | 25 (30.5) | 20 (31.7) | ... | 0.98 |
| *GT* | 36 (43.9) | 28 (44.4) | ... |  |
| *TT* | 21 (25.6) | 15 (23.8) | ... |  |
| ***fIFNG+1082*** |  |  |  |  |
| *AA* | 33 (40.2) | 24 (38.1) | ... | 0.85 |
| *AG* | 30 (36.6) | 26 (41.3) | ... |  |
| *GG* | 19 (23.2) | 13 (20.6) | ... |  |
| ***fIFNG+1133*** |  |  |  |  |
| *GG* | 67 (81.7) | 51 (81.0) | ... | 1.0 |
| *GC* | 13 (15.9) | 10 (15.9) | ... |  |
| *CC* | 2 (2.4) | 2 (3.2) | ... |  |
| ***fIFNG+1207*** |  |  |  |  |
| *CC* | 23 (28.0) | 11 (17.5) | ... | 0.06 |
| *CT* | 27 (26.8) | 33 (52.4) | ... |  |
| *TT* | 32 (39.0) | 19 (30.2) | ... |  |
