# Supplementary material for: Identification and genotyping of feline infectious peritonitis-associated single nucleotide polymorphisms in the feline interferon-γ gene
Source: Vet Res. 2014 May 21;45(1):57. doi: 10.1186/1297-9716-45-57 (PMC4041894; doi:10.1186/1297-9716-45-57)
Supplement: Additional file 2 — Frequencies of various alleles and their associations with the outcome of FCoV infection. The T allele at position +428 was significantly associated with the resistance of FIP. [file 1297-9716-45-57-S2.docx]

**Additional file 2 Frequencies of various alleles and their associations with the outcome of FCoV infection.**

| SNP | Control  Number (%) | FIP  Number (%) | OR (95% CI) | *P* |
| --- | --- | --- | --- | --- |
| ***fIFNG+230*** |  |  |  |  |
| Allele *T* | 153 (93.3) | 116 (92.1) | ... | 0.82 |
| Allele *C* | 11 (6.7) | 10 (7.9) | ... |  |
| ***fIFNG+253*** |  |  |  |  |
| Allele *G* | 115 (70.1) | 79 (62.7) | ... | 0.21 |
| Allele *C* | 49 (29.9) | 47 (33.3) | ... |  |
| ***fIFNG+308*** |  |  |  |  |
| Allele *A* | 153 (93.3) | 116 (92.1) | ... | 0.82 |
| Allele *C* | 11 (6.7) | 10 (7.9) | ... |  |
| ***fIFNG+333*** |  |  |  |  |
| Allele *A* | 126 (76.8) | 97 (77.0) | ... | 1.00 |
| Allele *G* | 38 (23.2) | 29 (23.0) | ... |  |
| ***fIFNG+401*** |  |  |  |  |
| Allele *T* | 112 (68.3) | 80 (63.5) | ... | 0.45 |
| Allele *C* | 52 (31.7) | 46 (36.5) | ... |  |
| ***fIFNG+408*** |  |  |  |  |
| Allele *T* | 112 (68.3) | 80 (63.5) | ... | 0.45 |
| Allele *C* | 52 (31.7) | 46 (36.5) | ... |  |
| ***fIFNG+428*** |  |  |  |  |
| Allele *C* | 148 (90.2) | 122 (96.8) | 3.3 (1.1-10.1) | 0.03 |
| Allele *T* | 16 (9.8) | 4 (3.2) | Reference |  |
| ***fIFNG+468*** |  |  |  |  |
| Allele *C* | 100 (61.0) | 72 (57.1) | ... | 0.55 |
| Allele *T* | 64 (39.0) | 54 (42.9) | ... |  |
| ***fIFNG+523*** |  |  |  |  |
| Allele *C* | 97 (59.1) | 77 (61.1) | ... | 0.81 |
| Allele *T* | 67 (40.9) | 49 (38.9) | ... |  |
| ***fIFNG+524*** |  |  |  |  |
| Allele *G* | 146 (89.0) | 112 (88.9) | ... | 1.0 |
| Allele *A* | 18 (11.0) | 14 (11.1) | ... |  |
| ***fIFNG+564*** |  |  |  |  |
| Allele *A* | 150 (91.5) | 113 (89.7) | ... | 0.68 |
| Allele *G* | 14 (8.2) | 13 (10.3) | ... |  |
| ***fIFNG+686*** |  |  |  |  |
| Allele *A* | 156 (95.1) | 120 (95.2) | ... | 1.0 |
| Allele *G* | 8 (4.9) | 6 (4.8) | ... |  |
| ***fIFNG+761*** |  |  |  |  |
| Allele *G* | 86 (52.4) | 68 (54.0) | ... | 0.81 |
| Allele *T* | 78 (47.6) | 58 (46.0) | ... |  |
| ***fIFNG+1082*** |  |  |  |  |
| Allele *A* | 96 (58.5) | 74 (58.7) | ... | 1.0 |
| Allele *G* | 68 (41.5) | 52 (41.3) | ... |  |
| ***fIFNG+1133*** |  |  |  |  |
| Allele *G* | 147 (89.6) | 112 (88.9) | ... | 0.85 |
| Allele *C* | 17 (10.4) | 14 (11.1) | ... |  |
| ***fIFNG+1207*** |  |  |  |  |
| Allele *C* | 91 (55.5) | 71 (56.3) | ... | 0.91 |
| Allele *T* | 73 (44.5) | 55 (43.7) | ... |  |
