# Supplementary material for: Identification and genotyping of feline infectious peritonitis-associated single nucleotide polymorphisms in the feline interferon-γ gene
Source: Vet Res. 2014 May 21;45(1):57. doi: 10.1186/1297-9716-45-57 (PMC4041894; doi:10.1186/1297-9716-45-57)
Supplement: Additional file 3 — Frequencies of various genotypes and their associations with the outcomes of type I FCoV infection. The heterologous genotypes of fIFNG + 401 and fIFNG + 408 were associated with the susceptibility to FIP in type I FCoV-infected cats. [file 1297-9716-45-57-S3.docx]

**Additional file 3 Frequencies of various genotypes and their associations with the outcomes of type I FCoV infection.**

| SNP | Control  Number (%) | FIP  Number (%) | OR (95% CI) | *P* |
| --- | --- | --- | --- | --- |
| ***fIFNG+230*** |  |  |  |  |
| *TT* | 73 (89.0) | 23 (79.3) | ... | 0.19 |
| *CT* | 7 (8.5) | 6 (20.7) | ... |  |
| *CC* | 2 (2.4) | 0 (0.0) | ... |  |
| ***fIFNG+253*** |  |  |  |  |
| *GG* | 43 (52.4) | 13 (44.8) | ... | 0.68 |
| *GC* | 29 (35.4) | 13 (44.8) | ... |  |
| *CC* | 10 (12.2) | 3 (10.3) | ... |  |
| ***fIFNG+308*** |  |  |  |  |
| *AA* | 73 (89.0) | 23 (79.3) | ... | 0.19 |
| *AC* | 7 (8.5) | 6 (20.7) | ... |  |
| *CC* | 2 (2.4) | 0 (0.0) | ... |  |
| ***fIFNG+333*** |  |  |  |  |
| *AA* | 50 (61.0) | 21 (72.4) | ... | 0.63 |
| *AG* | 26 (31.7) | 7 (24.1) | ... |  |
| *GG* | 6 (7.3) | 1 (3.4) | ... |  |
| ***fIFNG+401*** |  |  |  |  |
| *TT* | 43 (52.4) | 11 (37.9) | 0.4 (0.2-0.9) | 0.004 |
| *CT* | 26 (31.7) | 18 (62.1) | Reference |  |
| *CC* | 13 (15.9) | 0 (0.0) | n/a^a^ |  |
| ***fIFNG+408*** |  |  |  |  |
| *TT* | 43 (52.4) | 11 (37.9) | 0.4 (0.2-0.9) | 0.004 |
| *CT* | 26 (31.7) | 18 (62.1) | Reference |  |
| *CC* | 13 (15.9) | 0 (0.0) | n/a^a^ |  |
| ***fIFNG+428*** |  |  |  |  |
| *CC* | 66 (80.5) | 26 (89.7) | ... | 0.39 |
| *CT* | 16 (19.5) | 3 (10.3) | ... |  |
| *TT* | 0 (0.0) | 0 (0.0) | ... |  |
| ***fIFNG+468*** |  |  |  |  |
| *CC* | 30 (36.6) | 7 (24.1) | ... | 0.37 |
| *CT* | 40 (48.8) | 15 (51.7) | ... |  |
| *TT* | 12 (14.6) | 7 (24.1) | ... |  |
| ***fIFNG+523*** |  |  |  |  |
| *CC* | 34 (41.5) | 9 (31.0) | ... | 0.18 |
| *CT* | 29 (35.4) | 16 (55.2) | ... |  |
| *TT* | 19 (23.2) | 4 (13.8) | ... |  |
| ***fIFNG+524*** |  |  |  |  |
| *GG* | 66 (80.5) | 22 (75.9) | ... | 0.82 |
| *GA* | 14 (17.1) | 6 (20.7) | ... |  |
| *AA* | 2 (2.4) | 1 (3.4) | ... |  |
| ***fIFNG+564*** |  |  |  |  |
| *AA* | 71 (86.6) | 23 (79.3) | ... | 0.25 |
| *AG* | 8 (9.8) | 6 (20.7) | ... |  |
| *GG* | 3 (3.7) | 0 (0.0) | ... |  |
| ***fIFNG+686*** |  |  |  |  |
| *AA* | 75 (91.5) | 25 (86.2) | ... | 0.59 |
| *AG* | 6 (7.3) | 4 (13.8) | ... |  |
| *GG* | 1 (1.2) | 0 (0.0) | ... |  |
| ***fIFNG+761*** |  |  |  |  |
| *GG* | 25 (30.5) | 10 (34.5) | ... | 0.86 |
| *GT* | 36 (43.9) | 11 (37.9) | ... |  |
| *TT* | 21 (25.6) | 8 (27.6) | ... |  |
| ***fIFNG+1082*** |  |  |  |  |
| *AA* | 33 (40.2) | 11 (37.9) | ... | 0.70 |
| *AG* | 30 (36.6) | 13 (44.8) | ... |  |
| *GG* | 19 (23.2) | 5 (17.2) | ... |  |
| ***fIFNG+1133*** |  |  |  |  |
| *GG* | 67 (81.7) | 23 (79.3) | ... | 1.00 |
| *GC* | 13 (15.9) | 5 (17.2) | ... |  |
| *CC* | 2 (2.4) | 1 (3.4) | ... |  |
| ***fIFNG+1207*** |  |  |  |  |
| *CC* | 23 (28.0) | 4 (13.8) | ... | 0.06 |
| *CT* | 27 (26.8) | 17 (58.6) | ... |  |
| *TT* | 32 (39.0) | 8 (27.6) | ... |  |

^a^ not available.
