# Supplementary material for: Identification and genotyping of feline infectious peritonitis-associated single nucleotide polymorphisms in the feline interferon-γ gene
Source: Vet Res. 2014 May 21;45(1):57. doi: 10.1186/1297-9716-45-57 (PMC4041894; doi:10.1186/1297-9716-45-57)
Supplement: Additional file 4 — Frequencies of various alleles and their associations with the outcomes of type I FCoV infection. No allele of the analyzed SNP was associated with the occurrence of FIP in type I FCoV-infected cats. [file 1297-9716-45-57-S4.docx]

**Additional file 4 Frequencies of various alleles and their associations with the outcomes of type I FCoV infection.**

| SNP | Control  Number (%) | FIP  Number (%) | OR (95% CI) | *P* |
| --- | --- | --- | --- | --- |
| ***fIFNG+230*** |  |  |  |  |
| Allele *T* | 153 (93.3) | 52 (89.7) | ... | 0.39 |
| Allele *C* | 11 (6.7) | 6 (10.3) | ... |  |
| ***fIFNG+253*** |  |  |  |  |
| Allele *G* | 115 (70.1) | 39 (67.2) | ... | 0.74 |
| Allele *C* | 49 (29.9) | 19 (32.8) | ... |  |
| ***fIFNG+308*** |  |  |  |  |
| Allele *A* | 153 (93.3) | 52 (89.7) | ... | 0.39 |
| Allele *C* | 11 (6.7) | 6 (10.3) | ... |  |
| ***fIFNG+333*** |  |  |  |  |
| Allele *A* | 126 (76.8) | 49 (84.5) | ... | 0.26 |
| Allele *G* | 38 (23.2) | 9 (15.5) | ... |  |
| ***fIFNG+401*** |  |  |  |  |
| Allele *T* | 112 (68.3) | 40 (69.0) | ... | 1.00 |
| Allele *C* | 52 (31.7) | 18 (31.0) | ... |  |
| ***fIFNG+408*** |  |  |  |  |
| Allele *T* | 112 (68.3) | 40 (69.0) | ... | 1.00 |
| Allele *C* | 52 (31.7) | 18 (31.0) | ... |  |
| ***fIFNG+428*** |  |  |  |  |
| Allele *C* | 148 (90.2) | 55 (94.8) | ... | 0.41 |
| Allele *T* | 16 (9.8) | 3 (5.2) | ... |  |
| ***fIFNG+468*** |  |  |  |  |
| Allele *C* | 100 (61.0) | 29 (50.0) | ... | 0.16 |
| Allele *T* | 64 (39.0) | 29 (50.0) | ... |  |
| ***fIFNG+523*** |  |  |  |  |
| Allele *C* | 97 (59.1) | 34 (58.6) | ... | 1.00 |
| Allele *T* | 67 (40.9) | 24 (41.4) | ... |  |
| ***fIFNG+524*** |  |  |  |  |
| Allele *T* | 146 (89.0) | 50 (86.2) | ... | 0.64 |
| Allele *C* | 18 (11.0) | 8 (13.8) | ... |  |
| ***fIFNG+564*** |  |  |  |  |
| Allele *A* | 150 (91.5) | 52 (89.7) | ... | 0.79 |
| Allele *G* | 14 (8.2) | 6 (10.3) | ... |  |
| ***fIFNG+686*** |  |  |  |  |
| Allele *A* | 156 (95.1) | 54 (93.1) | ... | 0.74 |
| Allele *G* | 8 (4.9) | 4 (6.9) | ... |  |
| ***fIFNG+761*** |  |  |  |  |
| Allele *G* | 86 (52.4) | 31 (53.4) | ... | 1.00 |
| Allele *T* | 78 (47.6) | 27 (46.6) | ... |  |
| ***fIFNG+1082*** |  |  |  |  |
| Allele *A* | 96 (58.5) | 35 (60.3) | ... | 0.88 |
| Allele *G* | 68 (41.5) | 23 (39.7) | ... |  |
| ***fIFNG+1133*** |  |  |  |  |
| Allele *G* | 147 (89.6) | 51 (87.9) | ... | 0.81 |
| Allele *C* | 17 (10.4) | 7 (12.1) | ... |  |
| ***fIFNG+1207*** |  |  |  |  |
| Allele *C* | 91 (55.5) | 33 (57.0) | ... | 0.88 |
| Allele *T* | 73 (44.5) | 25 (42.7) | ... |  |
