# Supplementary material for: Identification and genotyping of feline infectious peritonitis-associated single nucleotide polymorphisms in the feline interferon-γ gene
Source: Vet Res. 2014 May 21;45(1):57. doi: 10.1186/1297-9716-45-57 (PMC4041894; doi:10.1186/1297-9716-45-57)
Supplement: Additional file 5 — Concentration of IFN-γ in the plasma samples of FIP cats carrying different genotypes at positions + 401, +408, and +428 on fIFNG. All the FIP cats carrying the CT genotype at position +428 were positive for the detection of plasma IFN-γ. [file 1297-9716-45-57-S5.docx]

**Additional file 5 Concentration of IFN-γ in the plasma samples of FIP cats carrying different genotype at position+401, +408, and +428 on *fIFNG.***

| Case No. | Genotype | | IFN-γ concentration (pg/mL)^a^ |
| --- | --- | --- | --- |
|  | +401/+408 | +428 |  |
| 7 | TT | CC | ND^b^ |
| 10 | CT | CC | ND |
| 13 | CT | CC | ND |
| 14 | CT | CT | > 8000 |
| 15 | TT | CC | ND |
| 17 | TT | CT | 51.5 |
| 22 | CT | CC | ND |
| 31 | CT | CC | ND |
| 39 | TT | CC | ND |
| 40 | CC | CC | ND |
| 53 | TT | CC | ND |
| 57 | CT | CC | ND |
| 58 | TT | CC | ND |
| 68 | TT | CC | ND |
| 69 | CC | CT | 138.1 |

^a^ plasma samples were collected at the day of presenting.

^b^ no detectable concentration of IFN-γ.
